# Earlier and more frequent occupation of breeding sites during the non‐breeding season increases breeding success in a colonial seabird

**DOI:** 10.1002/ece3.9213

**Published:** 2022-09-09

**Authors:** Sophie Bennett, Mike P. Harris, Sarah Wanless, Jonathan A. Green, Mark A. Newell, Kate R. Searle, Francis Daunt

**Affiliations:** ^1^ UK Centre for Ecology & Hydrology Edinburgh Midlothian UK; ^2^ School of Environmental Sciences University of Liverpool Liverpool UK

**Keywords:** breeding timing, common murre, non‐breeding behavior, productivity, site defense hypothesis, site quality, time‐lapse photography

## Abstract

Competition for high‐quality breeding sites in colonial species is often intense, such that individuals may invest considerable time in site occupancy even outside the breeding season. The site defense hypothesis predicts that high‐quality sites will be occupied earlier and more frequently, consequently those sites will benefit from earlier and more successful breeding. However, few studies relate non‐breeding season occupancy to subsequent breeding performance limiting our understanding of the potential life‐history benefits of this behavior. Here, we test how site occupancy in the non‐breeding season related to site quality, breeding timing, and breeding success in a population of common guillemots *Uria aalge*, an abundant and well‐studied colonially breeding seabird. Using time‐lapse photography, we recorded occupancy at breeding sites from October to March over three consecutive non‐breeding seasons. We then monitored the successive breeding timing (lay date) and breeding success at each site. On average, sites were first occupied on the 27th October ± 11.7 days (mean ± SD), subsequently occupied on 46 ± 18% of survey days and for 55 ± 15% of the time when at least one site was occupied. Higher‐quality sites, sites with higher average historic breeding success, were occupied earlier, more frequently and for longer daily durations thereafter. Laying was earlier at sites that were occupied more frequently and sites occupied earlier were more successful, supporting the site defense hypothesis. A path analysis showed that the return date had a greater or equal effect on breeding success as lay date. Pair level occupancy had no effect on breeding timing or success. The clear effect of non‐breeding occupancy of breeding sites on breeding timing and success highlights the benefits of this behavior on demography in this population and the importance of access to breeding sites outside the breeding season in systems where competition for high‐quality sites is intense.

## Graphical abstract

The relationship between occupancy of breeding sites in the non‐breeding season and subsequent breeding success is not well‐understood despite having likely impacts on population fitness. We used time‐lapse photography to record occupancy throughout the non‐breeding period, October–March, in a population of common guillemots to investigate how site occupancy related to site quality, timing of breeding, and breeding success. High‐quality sites were occupied earlier and more often with positive consequences for breeding timing and success and highlighting the benefits of this behavior on demography in this population and the importance of access to breeding sites outside of the breeding season in systems where competition for high‐quality sites is intense.

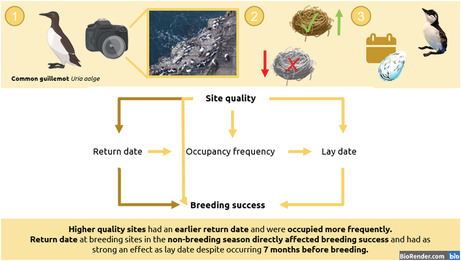

## 1 INTRODUCTION

In heterogeneous environments, breeding sites may differ in their physical properties, such as the protection they provide from harsh weather, or in their proximity to resources such as food (Harris et al., 1997; Pettorelli et al., 2001). Sites that have more favorable attributes may be of higher quality, offering fitness benefits to individuals breeding at them, for example, increased breeding success (Sergio & Newton, 2003) and/or likelihood of gaining a mate (Eckerle & Thompson, 2006). Consequently, where individuals can discern site quality, the highest‐quality sites will be preferentially occupied, as per the predictions of the site defense hypothesis. This process can lead to intense competition for access to sites of higher quality (Kokko et al., 2004). Furthermore, the ability of individuals to allocate more time to site occupancy can have a secondary benefit of strengthening pair bonds through joint occupation of sites (Beck et al., 2020), and in defending breeding partners from potential rival mates with further potential benefits for breeding success (Lemmon et al., 1997). Consequently, when competition for sites and mates is particularly fierce, as in colonial species, individuals may benefit from investing time and/or energy into site defense (Harrison et al., 2011).

In many seasonally breeding species, earlier occupation of breeding sites leads to more successful breeding (Aebischer et al., 1996). Individuals that commence site occupation earlier may occupy and defend higher‐quality sites for themselves and their breeding partner(s), which offer a higher likelihood of successful breeding (Forstmeier, 2002). In turn, individuals occupying sites earlier may also breed earlier (Morrison et al., 2019). Earlier breeding relative to conspecifics typically then leads to improved breeding success (Hatchwell, 1991), which can arise through, for example, optimal overlap with peak food abundance (Lepage et al., 2000). Due to the potential for both direct (via use of a high‐quality site) and indirect benefits (via early commencement of breeding), in some instances, breeding sites may be defended intermittently or continuously throughout the non‐breeding season (Crowther et al., 2018; Harris & Wanless, 2016). However, few studies have quantified variation in investment in site defense in the non‐breeding season, and how this relates to subsequent breeding timing and success. As a result, we lack a clear understanding of whether breeding sites with relatively high investment in non‐breeding site occupancy show improved subsequent breeding performance, and whether these benefits are realized via the earlier and/or more frequent occupancy of high‐quality sites.

The common guillemot *Uria aalge* (hereafter, guillemot) is an iteroparous colonially breeding seabird with a circumpolar breeding distribution spanning 36°0′N–78°0′N (Ainley et al., 2021). Individuals in many populations in the southern part of the breeding range return to occupy their breeding sites during the non‐breeding season, in the months between October and March (Harris & Wanless, 2016; Mudge et al., 1987; Sinclair, 2018). Previous work has shown that in the autumn, guillemots occupy sites of higher quality, that is, those that had previously been more successful, earlier and more frequently, and these sites were more successful the following breeding season (Harris & Wanless, 1989). However, site occupancy patterns have not been quantified throughout the non‐breeding season, which is a fundamental step to obtaining a comprehensive understanding of the effects of site occupancy on breeding success the following breeding season. Furthermore, it is unclear whether such benefits act directly on breeding success or indirectly via timing of breeding and whether there are additional effects of site occupancy on breeding when both members of a breeding pair are present.

Here, we use data collected by time‐lapse photography throughout the non‐breeding season to quantify timing, frequency, and duration of non‐breeding occupancy in a population of guillemots breeding on the Isle of May, south‐east Scotland. We collected these data in three consecutive years at breeding sites and followed their subsequent breeding success. First, we tested for evidence of the site defense hypothesis by examining whether sites of higher quality were occupied earlier, more frequently, and for longer daily duration (hypothesis 1). Second, we tested whether sites that were occupied earlier, more frequently, and for longer were bred at earlier the following breeding season (hypothesis 2a), and had higher breeding success (hypothesis 2b). Third, we tested whether occupancy directly affected breeding success, or whether any effects were indirect and sequential such that site quality affected return date, then occupancy frequency, lay date, and ultimately breeding success (hypothesis 3). We investigated the three hypotheses in situations when only one individual, one or two individuals, or two individuals occupied a site to test whether any effects were dependent on the number of individuals present at the site.

## 2 MATERIALS AND METHODS

The study was carried out on the Isle of May National Nature Reserve in the Firth of Forth, Scotland (56° 11′N, 02°33′W) from 2017 to 2020. We collected data on site occupancy during the non‐breeding season, timing of breeding, and breeding success in two areas (subcolony 1 and subcolony 2) of the large guillemot breeding colony on the island (14,902 breeding pairs in 2018 [Outram & Steel, 2018]). Both subcolonies were located on the west side of the island and were c. 60 m apart but not in line of sight of each other. Subcolony 1 had a fragmented structure with many small ledges, typically <20 cm wide; subcolony 2 had one large, broad ledge, c. 2 m × 1 m, and a number of smaller ledges, <1 m wide, see Appendix 1.

### 2.1 Monitoring breeding site occupancy in the non‐breeding season

We used time‐lapse photography to quantify breeding site occupancy during the non‐breeding season. We placed DSLR cameras in waterproof housings at each subcolony, adjacent to the vantage points used by observers to make breeding observations (c. 8 m away from subcolony 1 and c. 3 m away from subcolony 2; for more information on technical setup see Appendix 2). The cameras were installed in late September several weeks before the first birds were expected to return to the breeding sites following their postbreeding exodus. Female non‐breeding site occupancy decreases markedly in the period from early April until laying (Wanless & Harris, 1986). We therefore defined the non‐breeding season as beginning when the first bird(s) returned to the colony, and ending at the end of March. Occupancy data were collected for subcolony 1 only for the non‐breeding season of 2017/18, and for both subcolonies in 2018/19, and 2019/20. External timers triggered the cameras to take an image every 30 min in 2017/18 and every 15 min in 2018/19, and 2019/20. The different sampling regimes had no impact on any of our results or conclusions (Appendix 3). The cameras did not have night vision, but it was possible to determine that birds were absent from the colony overnight on moonlit nights, and just before sunrise and after sunset when sufficient light remained.

### 2.2 Image scoring

Using the time‐lapse images (*n* = 83,834), we recorded breeding site occupancy in both subcolonies. We defined a breeding site as the small area of a cliff ledge, ~10 cm × 10 cm where a pair later incubated an egg. To ensure consistency when assigning birds to sites, we took images of both subcolonies during the preceding breeding season from the same vantage points and marked the locations of pairs to produce breeding site maps, assigning each a unique ID. We then recorded whether zero, one, or two birds were present at each of these sites for each time‐lapse image using the maps as a reference (Appendix 1). After each breeding season, we reviewed the images from the previous non‐breeding season and retrospectively recorded the occupancy patterns at those sites that had not been bred at previously. In subcolony 1, we monitored 26–29 sites each year, and in subcolony 2, we monitored 51–54 sites (Table 1).

**TABLE 1:** The number of sites monitored for non‐breeding occupancy and breeding observations

Year	Subcolony	non‐breeding observations		Breeding observations	
		Total sites followed	Sampling days	Lay date	Breeding success
2017/18	1	26	207	26	26
2018/19	1	27	154	27	27
	2	51	177	54	54
2019/20	1	29	174	29	29
	2	54	118	50	19

During the three study years, there were sporadic periods when we were unable to score the images for site occupancy for some, or all, sites in a subcolony due to fog or loss of battery power (see Appendix 4 for dates and subcolonies affected). By considering the key measures of occupancy (number of days after return date and occupancy duration on each day) as proportional values, we minimized the impact of any data gaps.

### 2.3 Breeding timing and success

We made detailed observations of both subcolonies at least once a day from before the first egg was laid in late April until after the last chick fledged in mid‐July (Harris & Wanless, 1988) to determine timing of breeding, the ordinal date that an egg was laid at each site (lay date), and breeding success for the majority of sites (Table 1). We made our observations for subcolony 1 from a permanent hide, and those for subcolony 2 from a vantage point overlooking the subcolony. We then recorded the lay date at each site as the first day that an egg was seen by an observer. As guillemots only raise one chick a year, we considered a breeding attempt to be successful if a chick reached a minimum fledging age of 15 days unless there was evidence to the contrary (Harris et al., 2020). In 2020, we had to predominantly use images from cameras instead of direct observations to collect the majority of breeding data due to limited access to the study site during the COVID‐19 pandemic (details in Appendix 5), a method which has successfully been used to monitor both breeding phenology and success in other seabird species (Hinke et al., 2018).

### 2.4 Site quality measures

In guillemots, physical characteristics of breeding sites influence breeding success (Birkhead, 1977; Harris et al., 1997). Sites of higher quality are preferentially occupied during the breeding season in a density‐dependent manner and have a higher likelihood of a successful breeding outcome. This has been observed in two separate analyses of our study population (Bennett et al., 2022; Kokko et al., 2004). Hence, for subcolony 1, we used the average breeding success of a site based on data collected from 1981 to 2016 as a measure of site quality. The average breeding success of a site was the total number of successful breeding attempts divided by the total number of breeding attempts at a site. This measure of site quality is not entirely separable from potential effects of the quality of individuals breeding at sites, a long‐standing challenge in studies of this kind (Germain & Arcese, 2014). However, as the direct effects of physical site characteristics on breeding sites have been previously established in our study system (Harris et al., 1997), we are confident that this measure underpins effects of site quality. We were unable to include any measure of site quality for subcolony 2 because we did not have data on physical characteristics or long‐term data on breeding success.

### 2.5 Data treatment

To check whether individuals only occupied the site where they bred the following breeding season, we recorded the site occupied and color combination of any ringed birds in camera photographs (*n* = 29 birds subcolony 1, *n* = 37 subcolony 2). In the vast majority of instances (>99.3%, *n* = 3485 observations), birds were observed on their future breeding site. This supports earlier observations that in the non‐breeding season individuals only occupy the site where they subsequently breed (Harris & Wanless, 1989). We therefore assumed that all occupancy measures at each site represented individuals that subsequently bred at that site.

Camera images were used to quantify three occupancy measures: (1) the ordinal first date on which one or two birds occupied a site (return date), (2) how frequently a site was occupied (the proportion of days one or two birds were present from the return date to the end of March; occupancy frequency), and (3) the daily duration of time spent at a site relative to occupancy of other sites in the subcolony as indicated by the number of images on each day that a site was occupied divided by the number of images on each day where at least one bird was present in the subcolony (relative time investment).

### 2.6 Statistical analyses

We used general and generalized linear mixed‐models to test all hypotheses. All continuous explanatory variables were standardized for each subcolony and year prior to modeling by subtracting the mean and dividing by the standard deviation for each subcolony for each year. We included a random term of “Site ID” in all models to accommodate site level variation, not included in our covariates. Unless stated otherwise, we also included a random effect of “Subcolony Year” (e.g., Subcolony 1 in 2017) to account for interannual and inter‐subcolony differences in occupancy, and/or breeding parameters that may arise from unmeasured environmental and individual factors.

### 2.7 Associations between occupancy measures

Prior to testing the effect of our three occupancy measures on timing of breeding and breeding success, we tested the associations between these measures. This was to establish whether sites occupied earlier were also those occupied more frequently during the non‐breeding season, and for longer each day, or whether these measures were independent of one another. To examine these relationships, we used two generalized linear mixed‐effects models with a binomial error structure and a logit link. In the first model, we tested whether those sites that were occupied earlier were occupied more frequently. The explanatory variable was return date, and our response variable was the occupancy frequency. In the second model, we tested whether sites that were occupied earlier and more frequently, were occupied for longer each day. Here, we included both the return date and the frequency with which a site was occupied as explanatory variables, and the relative time investment at a site each day as the response. We also included a two‐way interaction between return date and occupancy frequency to test whether the effect of occupancy frequency on the relative time investment at a site was intensified by returning earlier.

Hypothesis 1 Site quality and occupancy (site defense hypothesis).

We tested for evidence of the site defense hypothesis that a key motivation for birds to occupy the breeding site in the non‐breeding season is to defend a high‐quality breeding site (hypothesis 1). We predicted that higher‐quality sites would have an earlier return date, be occupied more frequently, and for longer during the day. For this analysis, we used only data from subcolony 1 (*n* = 20 sites, 19 with three years and, one with one year of data). We tested each of these occupancy measures in three separate general linear mixed‐effects model, each with site quality as our explanatory variable and the occupancy measure as the response. For the model with return date as the response, we used a Gaussian error structure (normality determined by quantile‐quantile (QQ plots) and two‐sided Kolmogorov–Smirnov tests (return date: *D* = 0.11, *p* = .09)). For the other two models, we used a binomial error distribution and a logit link. We included a fixed effect of “year” to test whether occupancy measures varied interannually.

Hypothesis 2 Occupancy and breeding.

We then quantified the effects of the three occupancy measures on lay date (hypothesis 2a), and breeding success (hypothesis 2b). First, we tested our hypothesis 2a that those sites that are first occupied earlier, more frequently, and for longer had an earlier lay date in the following breeding season. Here, the ordinal lay date for a site was our response variable with a Gaussian error structure (normality checked using Kolmogorov–Smirnov test: *D* = 0.11, *p* = .12 and *QQ* plots). Next, we tested our hypothesis 2b, that those sites that are occupied earlier, more frequently, and for longer had a higher likelihood of having a successful breeding attempt. Here, the breeding success of a site was our response variable, assuming a binomial error structure with a logit link (as breeding attempts were either successful, 1, or unsuccessful, 0). In both models, we included all two‐way and three‐way interactions to test whether any effect of occupying a site more frequently or for longer was intensified by occupying sites earlier than conspecifics.

In addition, we tested whether any effects of site occupancy on breeding were stronger when both members of a pair simultaneously occupied the site. We repeated all of the analyses and validation steps adopted in the main analysis involving occupancy by one or two birds, but restricting the occupancy data to when two birds were present at a site. The pair‐level analysis demonstrated the same relationships between occupancy measures and between site quality and occupancy measures. However, this pair‐level analysis differed from the main analysis; in that, there was a lack of an effect of pair‐level occupancy measures on breeding timing or success; full details of this analysis are in Appendix 6. Furthermore, we tested whether any relationships between site quality, occupancy and breeding timing and success were different using occupancy measures for when just one bird was present. In these tests, we found no significant differences from our main analysis. We present a summary of these tests in Appendix 7.

### 2.8 Model validation

We fitted models for hypotheses 1 and 2 using the R package “lme4” (Bates et al., 2001, p. 4). Where a model contained more than one explanatory variable, we tested all possible combinations of each term. We then selected the top model using Akaike's information criterion (AIC) to assess relative support in the data for each model employing a nested approach; where ΔAIC to the model with the next closest AIC was >2, we selected the model with the lowest AIC, (Burnham & Anderson, 1998). Alternatively, where the ΔAIC between two models was <2, we selected the most parsimonious, un‐nested model (Appendix 8). We derived 95% confidence intervals for model terms using the “confint” function in the R “stats” package (R Core Team, 2021). We considered fixed effects to be significant if their confidence intervals did not cross zero (Zuur et al., 2009). In top models, we then tested different random effect structures to determine which was most appropriate for our data. We ran four models with the same fixed effect structure but with either a random intercept, combined intercept and slope, a separate intercept and slope or just a random slope. We then determined which structure received the most support in the data through comparison of AIC values as for fixed effects (Appendix 9). We present results only for the most supported model in each case.

We inspected explanatory variables for autocorrelation and disregarded models where this exceeded >0.7 (Dormann et al., 2013), and inspected residual plots to ensure distributions were random. Means are presented ± standard deviations unless indicated otherwise. We carried out all statistical analysis in R version 3.6.1 (R Core Team, 2021) and extracted prediction values from models using the package “sjPlot”(Lüdecke, 2019).

Hypothesis 3 Occupancy as a driver of breeding success

**FIGURE 1:**
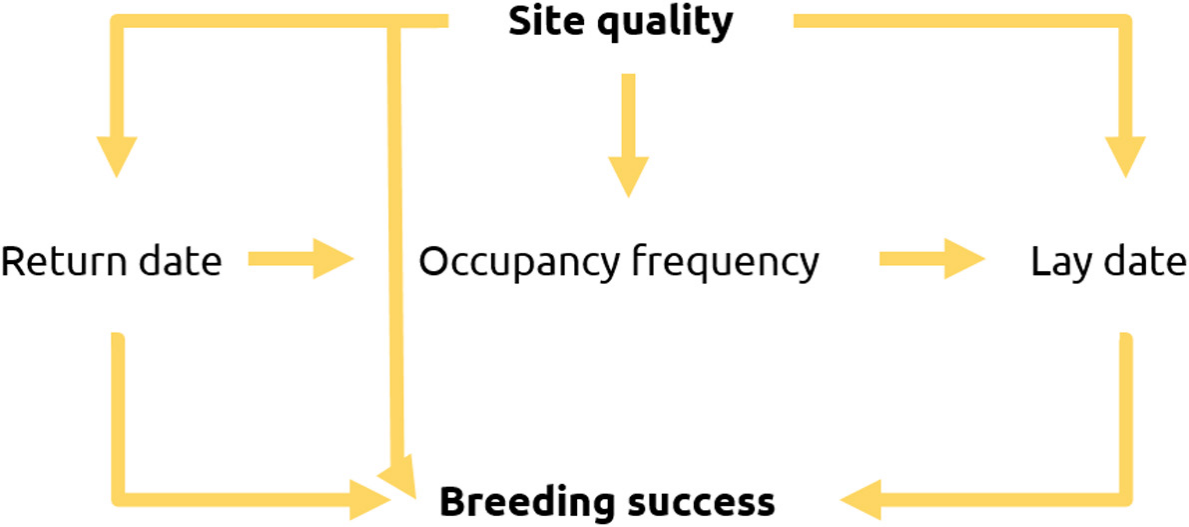
A conceptual diagram of the five pathways included in a path analysis. Arrows show the direction of pathways. All paths contained “Site quality” as a predictor and had “Breeding success” as the response.

Lastly, we used structural regression modeling via a path analysis to determine whether occupancy directly affected breeding success, or whether any effects were indirect and sequential such that site quality affected return date, then occupancy frequency, lay date, and ultimately breeding success. The relationships between site quality, lay date, and breeding success for guillemots are well established in the literature; breeding commences earlier at higher‐quality sites, and these sites have higher breeding success (Bennett et al., 2022; Kokko et al., 2004). However, the relationships between site quality, non‐breeding occupancy, and lay date and breeding success are not well characterized. Consequently, we used the findings from our tests of the first two hypotheses to inform the structure of the path analysis, constructing individual paths based on the evidence within these analyses for relationships between explanatory variables. This resulted in five possible pathways all containing breeding success as the response variable, and including site quality as a predictor (Figure 1).

### 2.9 Modeled pathways

**Table:** 

Breeding success ~ site quality
Breeding success ~ site quality + return date
Breeding success ~ site quality + lay date
Breeding success ~ site quality + occupancy frequency + lay date
Breeding success ~ site quality + return date + occupancy frequency + lay date

To test the support for each pathway, we used structural equation modeling in a Bayesian framework with three key model parts: data models which were the likelihood linking input data to the model parameters, process models linking the predictions from the model to the parameters and minimally informative prior distributions of parameters. For “Breeding success,” we assumed a Bernoulli probability, *p*, distribution with a logit function as input values were either “0” or “1,” as per:

$$\text{Breeding success}\sim \text{Bernoulli}\;\left(p,1\right)$$

For all other parameters, we assumed a normal probability distribution as per:

$$\text{Parameter}∼\text{Normal}\left(\mu ,\tau \right)$$

where *μ* is the mean estimated value for each observation, and *τ* is the precision. We then constructed regression models for each of the five pathways in JAGS using the R package “R2jags” (Su & Yajima, 2021). All regressions contained a random effect of “Site ID” to account for unmeasured site‐specific factors that may affect modeled relationships. Regressions took the form of:

$$\gamma_{i}=\alpha +\beta_{i}X_{i}+\varepsilon_{\text{Site}\;\mathrm{ID}}$$

where *γ*_*i*_ was the response for model *i*, *α* was the intercept, *β*_*i*_ was the path coefficient for variable *Χ*_*i*_ for model *i*, and *ε*_Site ID_ was a random effect of Site ID. Parameters *α* and *β*_*i*_ were both assigned minimally informative priors with a normal distribution with a mean of 0 and a precision of 0.001, *ε*_Site ID_ was assigned a minimally informative prior with a gamma distribution with a mean of 0 and a precision of 0.001. Before modeling, we standardized (mean‐centered and scaled) all variables. For this analysis, we included only data from subcolony 1 as we did not have site quality measures for sites in subcolony 2.

We ran the model with three chains, each with 200,000 iterations, a thinning interval of three and a burn in of 15,000. The model successfully converged under these parameters; the Gelman–Ruben statistics for all variables were between 1 and 1.05 (Brooks & Gelman, 1998), effective sample sizes were >400 and trace plots indicating good mixing of chains.

## 3 RESULTS

### 3.1 Patterns of occupancy

**FIGURE 2:**
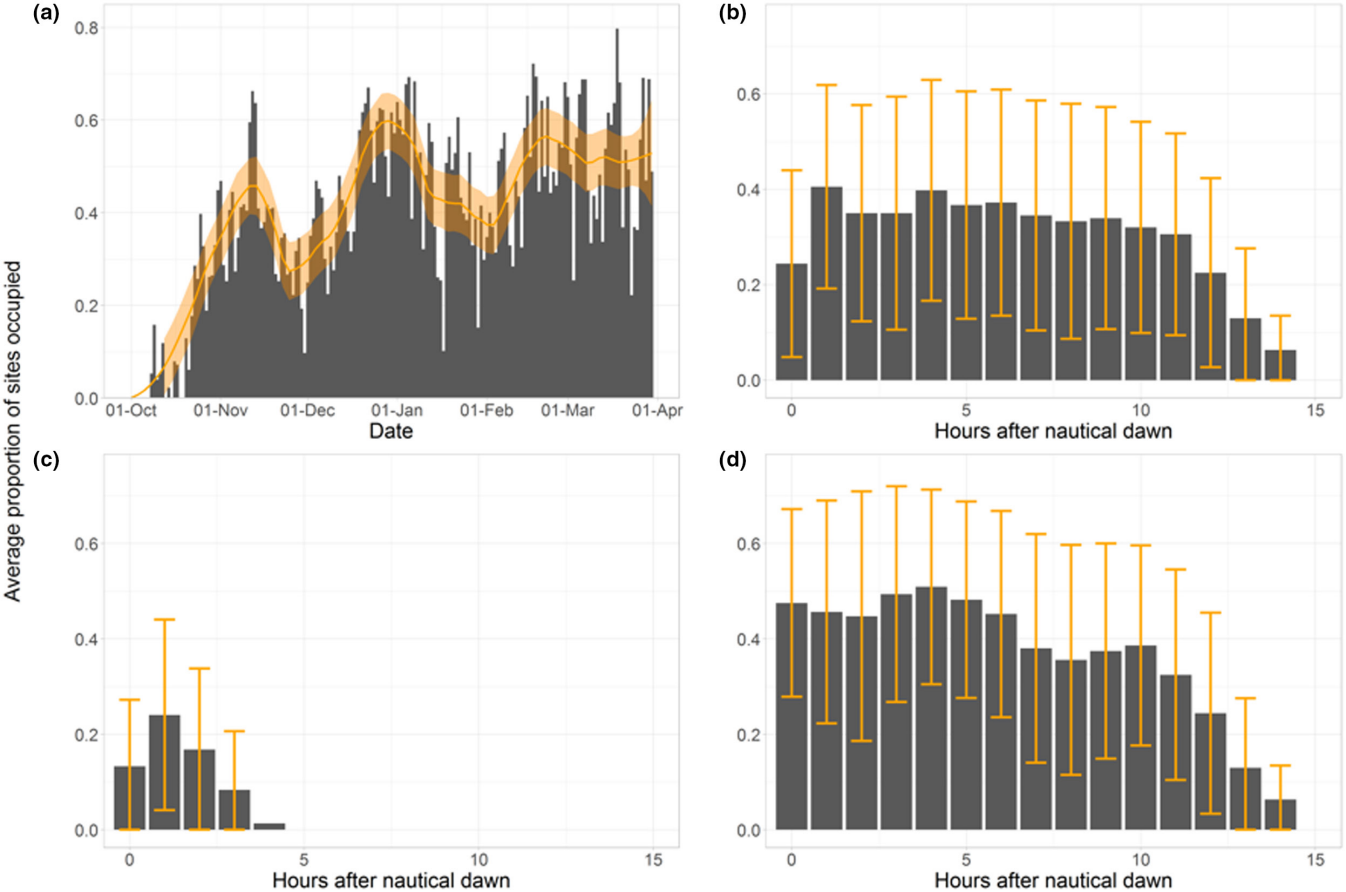
Patterns of occupancy averaged over two subcolonies and three non‐breeding seasons: (a) the proportion of breeding sites occupied in ≥one image/ day, and the proportion of sites occupied in each hour after sunrise in (b) all months, (c) October and, (d) march. In (a), the smoothed trend (orange line) and standard error (shaded area) are given. In (b–d), error bars indicate the standard deviation.

In both subcolonies and in all years, guillemots returned to the colony in mid‐October, with 80% of sites occupied at least once by October 31st (ordinal day = 305). Following initial return to the colony, the proportion of sites occupied generally increased until plateauing in ~mid‐March when ~50% of sites were occupied each day (Figure 2a). There were dips in occupancy in early December and early February. The diel pattern of occupancy was consistent throughout the year with occupancy peaking 1–2 h after nautical dawn and thereafter steadily declining until nautical dusk when no birds were present (Figures 2b–d). No overnight site occupancy was recorded.

### 3.2 Associations between occupancy measures

Overall, the mean date that a site was first occupied was October 27th ± 11.7 days (OD = 297). Sites were occupied for an average of 46 ± 18% of days during the non‐breeding period, and for 55 ± 15% of the time that a subcolony was occupied.

Sites occupied earlier in the autumn were also occupied more frequently (estimate = −0.02, 95% CI = −0.04, −0.01), but the relationship was weak, with a ten‐day difference in return rate resulting in a 2 ± 8% (±SE) increase in frequency. Those sites that were occupied earlier or more frequently were also occupied for longer on a given day (return date, estimate = −0.21, 95% CI = −0.34, −0.10, proportion of days, estimate = 0.46, 95% CI = 0.29, 0.65). These two effects had a positive interaction with one another, such that sites occupied 10 days earlier were occupied 5.1 ± 17% (±SE) longer, and for an additional 6 ± 14% (±SE) longer for each 10% increase in how frequently sites were occupied (estimate = −0.07, 95% CI = −0.034, −0.005) and vice versa. How early and frequently sites were occupied together explained almost half of model variance for how long sites were occupied (model 2: marginal *R* ^2^ = .41, conditional *R* ^2^ = .96). Return date alone explained a comparatively much smaller proportion of the variance in occupancy frequency (model 1: marginal *R* ^2^ = .01, conditional *R* ^2^ = .87). For both models, the most supported random effect structure contained a random intercept and slope for Subcolony year (Appendix 9).

Hypothesis 1 Site quality and occupancy.

**FIGURE 3:**
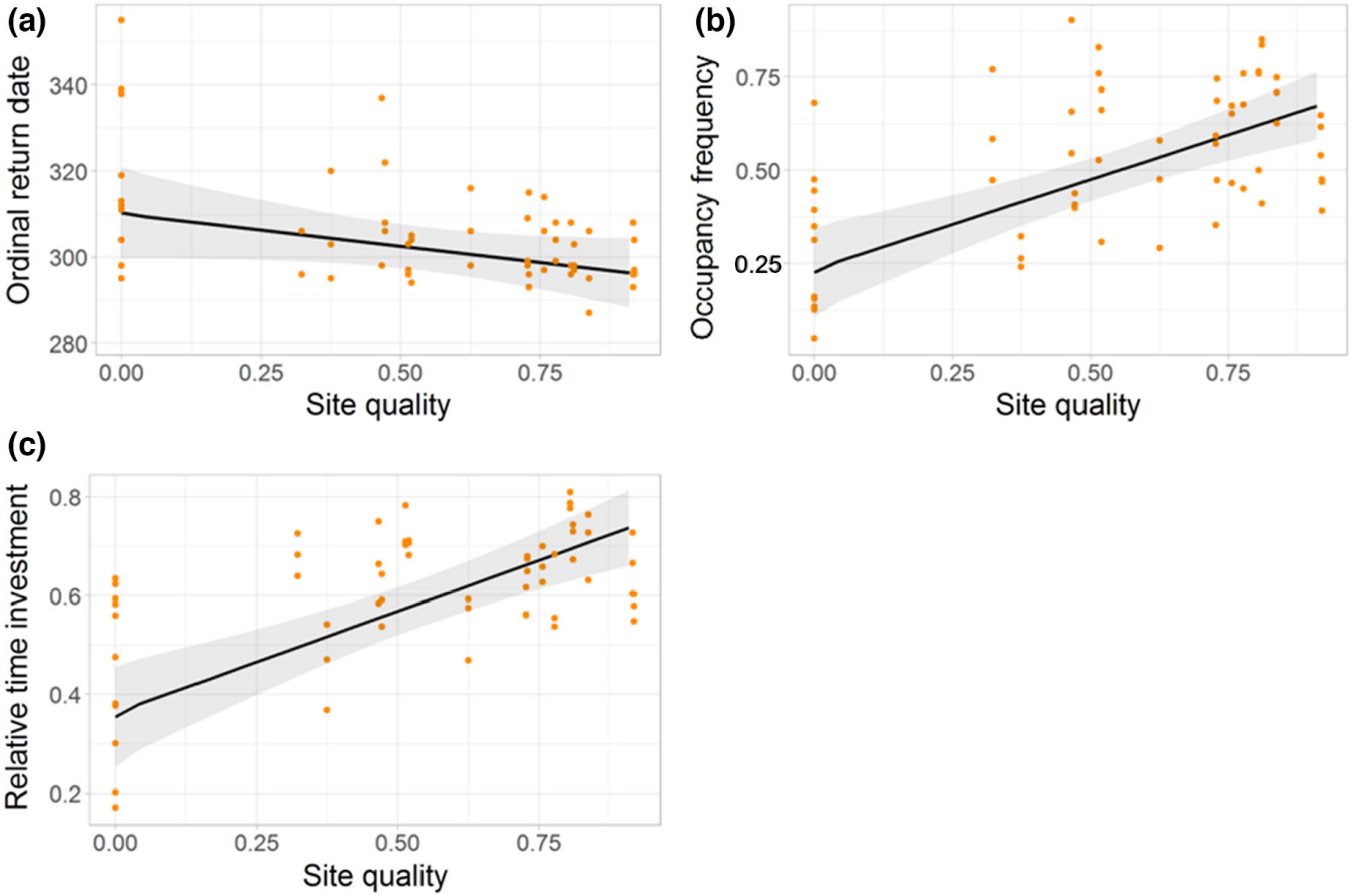
Relationship between a breeding site's quality and (a) return date, (b) occupancy frequency, and (c) relative time investment. Raw data (points) and GLMM model predictions (fitted line ±95% CI). *N* = 59.

Sites of higher quality were occupied earlier (model 1, estimate = −4.67, 95% CI = −9.72, −0.43, Figure 3a), more frequently (model 2, estimate = 0.82, 95% CI = 0.52, 1.12, Figure 3b), and for longer (model 3, estimate = 0.94, 95% CI = 0.63, 1.26, Figure 3c), see Table 2. For each 6% increase in quality, sites were occupied one day earlier, 3% more frequently, and for 3% longer. Return dates were generally earlier in 2019/20 than in 2017/18 and 2018/19 (model 1, estimate = 10.68, 95% CI = 4.41, 16.85), Furthermore, the strength of the relationship between site quality and how frequently a site was occupied, and the length of occupancy also varied between years; in 2019/20, the positive effect of site quality on these occupancy measures was weaker than in other years (see model 2, and model 3 and Appendix 10). Overall, the evidence supported hypothesis 1 that sites of higher quality were occupied earlier and more extensively, although the strength of this effect varied among years.

**TABLE 2:** General linear mixed‐effects model outputs assessing the effect of site quality on the (1) return date, and (2) the frequency that a site was occupied and (3) the relative time investment at a site

Model	Response variable	Fixed effects	Estimate	Standard error	95% confidence interval
1	Return date	**Intercept**	**301.97**	**2.59**	**297.03, 306.87**
		**Site quality**	**−4.67**	**2.66**	**−9.72, −0.43**
		Year			
		**2018/19**	**10.68**	**3.26**	**4.41, 16.85**
		2019/20	0.78	3.26	−5.49, 6.95
		Site quality*2018/19	−3.07	3.36	−9.41, 3.46
		Site quality*2019/20	−0.12	3.36	−6.46, 6.41
*Marginal R* ^*2*^ *= .31, Conditional R* ^*2*^ *= .45, n = 59*					
2	Occupancy frequency	**Intercept**	0.18	0.15	−0.12, 0.49
		**Site quality**	**0.82**	**0.15**	**0.52, 1.12**
		**Year**			
		**2018/19**	**0.32**	**0.06**	**0.20, 0.45**
		**2019/20**	**−0.79**	**0.08**	**−0.84, −0.54**
		Site quality*2018/19	**−0.44**	**0.07**	**−0.58, −0.31**
		Site quality*2019/20	**−0.35**	**0.08**	**−0.51, −0.18**
*Marginal R* ^*2*^ *= .55, Conditional R* ^*2*^ *= .96, n = 59*					
3	Relative time investment	**Intercept**	**−0.52**	**0.16**	**−0.83, −0.19**
		**Site quality**	**0.94**	**0.15**	**0.63, 1.26**
		**Year**			
		**2018/19**	**0.56**	**0.03**	**0.50, 0.61**
		**2019/20**	**−0.29**	**0.04**	**−0.36, −0.21**
		Site quality*2018/19	**−0.47**	**0.03**	**−0.53, −0.41**
		Site quality*2019/20	**−0.30**	**0.04**	**−0.38, −0.22**
*Marginal R* ^*2*^ *= .55, Conditional R* ^*2*^ *= .99, n = 59*					

*Note*: Significant terms, that is, those with 95% confidence intervals not overlapping zero, are in bold.

Hypothesis 2 Occupancy and breeding.

Hypothesis 2a Lay date.

**FIGURE 4:**
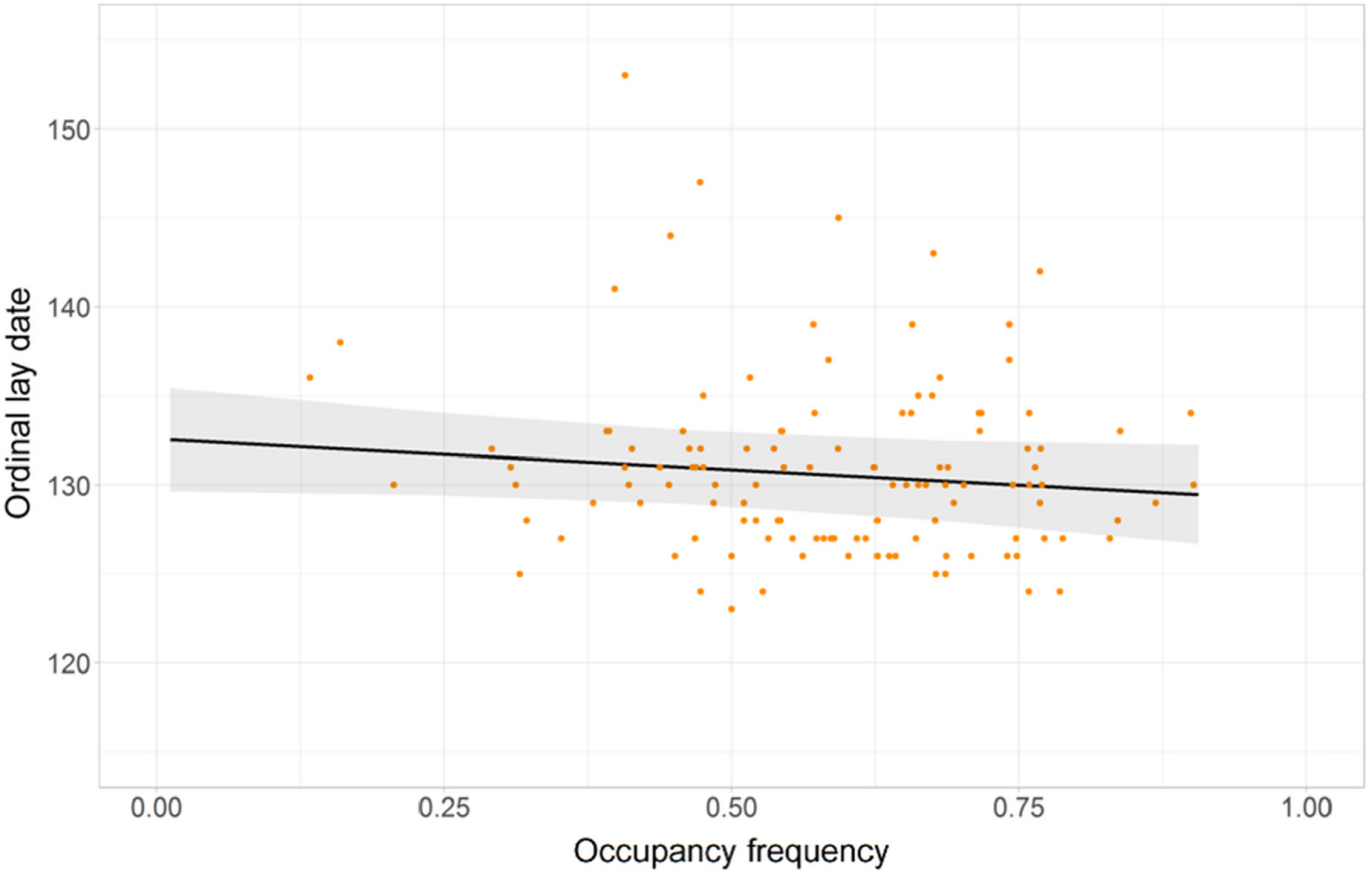
Effect of occupancy frequency on lay date. Raw data (points) and GLMM model predictions (fitted line ±95% CI). *N* = 120.

There was weak support for hypothesis 2a that sites that were occupied more frequently had an earlier lay date (estimate = −0.93, 95% CI = −1.97, −0.12, Figure 4) such that for each 24% increase in occupancy frequency, lay date was one day earlier. However, how early and how long a site was occupied were not retained in the most supported model (Table A11). The random terms “Subcolony Year” (*n* = 5) and “Site ID” (*n* = 58) contributed to a large part of model variance (marginal *R* ^2^ = .02, conditional *R* ^2^ = .54), reflecting the contributions of interannual and intersite variation in lay date.

Hypothesis 2b Breeding success.

**FIGURE 5:**
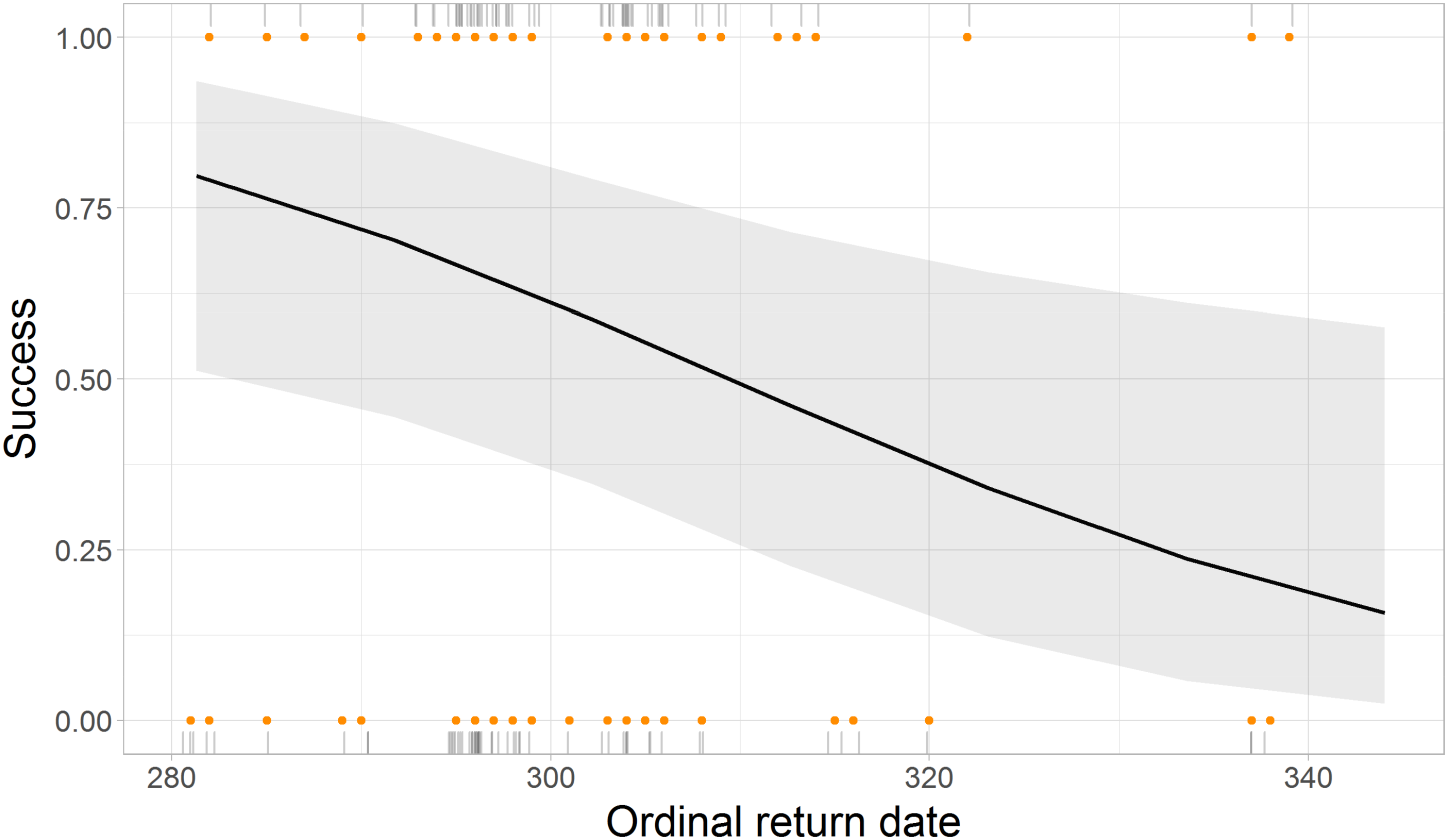
Generalized linear model predictions showing the relationship between the return date and breeding success. Raw data (points), accompanying rug plots (vertical gray bars), and GLMM model predictions (fitted line ±95% CI) are shown. In rug plots, darker bars indicate a higher density of raw data points. *N* = 123.

Sites were more likely to be successful when they were occupied earlier (estimate = −0.5, 95% CI = −1.06, −0.12, Figure 5) such that for each day earlier that sites were occupied their likelihood of success increased by up to 0.5%. How frequently and for how long a site was occupied were not retained in the final model (Table A12). However, models containing both how early and how frequently, and both how early and how long received partial support, but in neither case did additional occupancy measures have a significant effect (occupancy frequency: estimate = 0.25, 95% CI = −0.24, 0.78, relative time investment: estimate = 0.23, 95% CI = −0.29, 0.78). As with the lay date tests, only one of the occupancy measures had a clear effect on breeding, again providing partial support for hypothesis 2b, since sites that were occupied earlier were more likely to have a successful breeding outcome.

As with the tests on lay date, the random‐term component of “Subcolony Year” (*n* = 5), and “Site ID” (*n* = 59) in the model explained a large proportion of model variance (marginal *R* ^2^ = .06, conditional *R* ^2^ = .29). This result likely reflects the susceptibility of breeding outcome to extrinsic factors between years untested here.

Hypothesis 3 Occupancy as a driver of breeding success.

**FIGURE 6:**
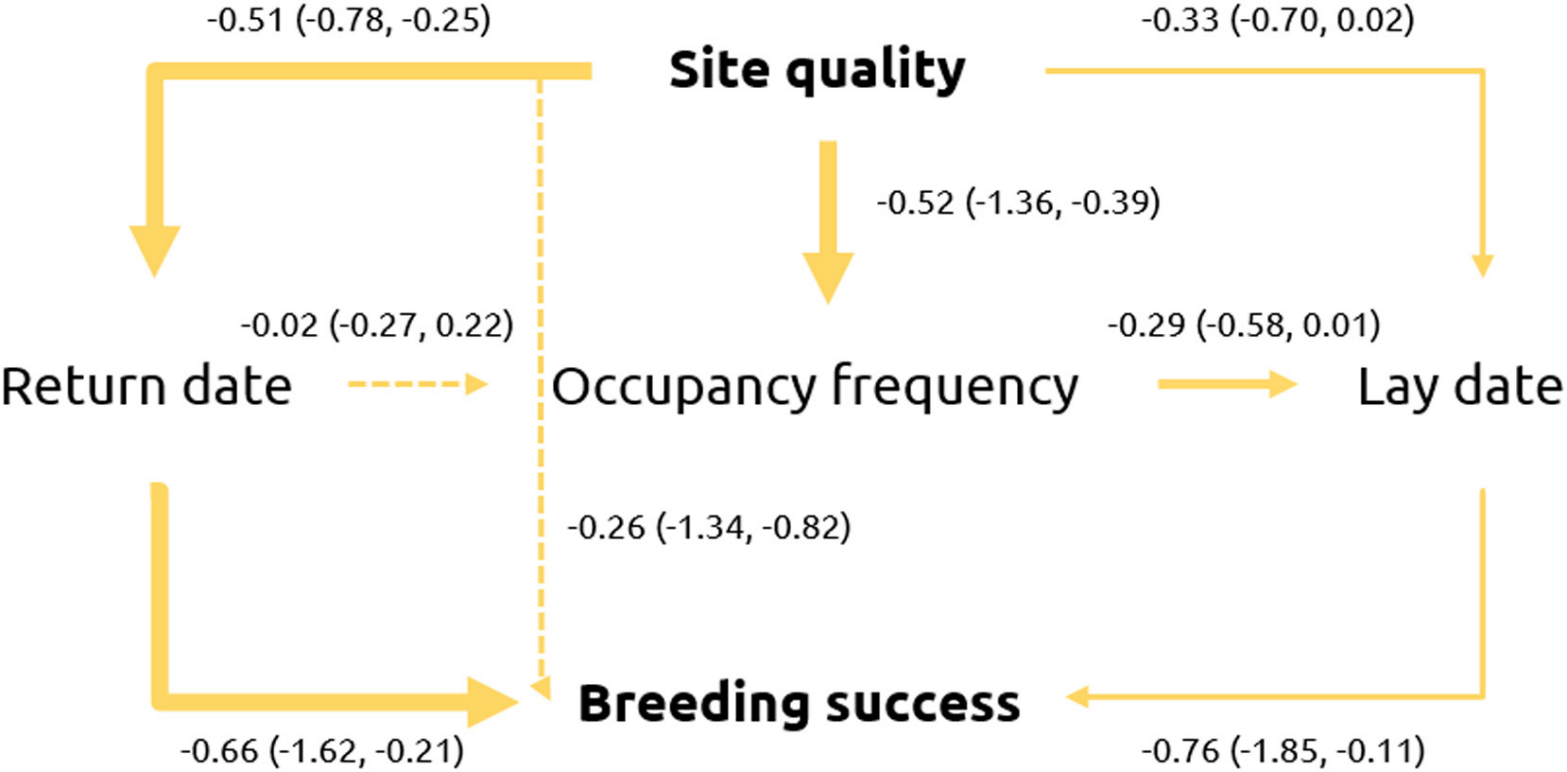
Path analysis diagram showing the relationships between site quality, occupancy measures, and breeding success. Values between variables are standardized estimates of the relationship between those variables (95% credible intervals). Lines indicate model support for an effect: Strong (thick), some (thin), none (dashed).

Breeding success was affected both directly and indirectly by site occupancy and lay date. The most‐supported pathway contained only site quality and return date (estimate: 0.33, 95% CI = −0.10, 0.92), 93.1% of posterior density greater than zero. The effect of return date on breeding success also had the most support of all pathway steps, such that 99.6% of the posterior was positive (Figure 6). However, the pathway containing site quality and lay date (estimate: 0.25, 95% CI = −0.06, 0.83), and the pathway containing site quality, occupancy frequency, and lay date (estimate: 0.11, 95% CI = −0.03, 0.41) received almost as much support, but with an effect size more than two times smaller (Table 3). Together, these results demonstrate that there is reasonable support for non‐breeding occupancy having both direct effects of return date on breeding success, and indirect effects whereby occupancy frequency affects breeding success via lay date, and that these effects are equally or more important than direct effects of lay date on breeding success. Thus, we found some support for our third hypothesis that breeding success would operate indirectly, and sequentially, through a pathway containing both occupancy measures and lay date. However, other pathways containing only some of these measures contained greater or equal support.

**TABLE 3:** Pathway coefficient estimates through which site quality, via lay date and/or non‐breeding occupancy, affects breeding success

Pathway	Standardized estimate (95% credible interval)	% of posterior with same sign as estimate
Breeding success ~ site quality + return date	0.33 (−0.10, 0.92)	93.1
Breeding success ~ site quality + lay date	0.25 (−0.06, 0.83)	92.5
Breeding success ~ site quality + occupancy frequency + lay date	0.11 (−0.03, 0.41)	92.3
Breeding success ~ site quality	−0.27 (−1.34, 0.82)	70.1
Breeding success ~ site quality + return date + occupancy frequency + lay date	0.002 (−0.04, 0.05)	56.0

*Note*: *N* observations = 50.

## 4 DISCUSSION

Using high‐resolution occupancy data, we found clear benefits of non‐breeding site occupancy for guillemots in terms of their subsequent breeding success. The proportion of sites occupied varied greatly across the non‐breeding period, but overall progressively more sites were occupied as the breeding season approached. Site quality was an important predictor of occupancy, with higher‐quality sites occupied earlier and more frequently supporting the site defense hypothesis (hypothesis 1). Our results show that occupancy had important effects on breeding performance; sites occupied earlier were more successful, and sites occupied more frequently had an earlier lay date, so supporting our second hypothesis that occupancy will have benefits for breeding. Finally, we found support for a direct effect of occupancy on breeding success, as well as evidence for a separate indirect effect of occupancy affecting breeding success via timing of breeding. Both the direct and the indirect effects of non‐breeding site occupancy received equal or greater support compared with the direct effect of timing of breeding on breeding success. This is in broad agreement with our third hypothesis which predicted that the positive effects of occupancy would sequentially affect timing of breeding and in turn breeding success. Occupancy of sites in the non‐breeding season can hence have a central role in the well‐established relationship between breeding timing and success.

Although the number of sites occupied in our study subcolonies varied greatly during the study period, the overall diel pattern of occupancy remained consistent. Site occupancy peaked within the first hours after nautical dawn and then gradually decreased throughout the day as birds left the colony presumably to forage (Dunn et al., 2020). The total number of sites occupied increased progressively from when the first birds returned in the autumn until upwards of 50% of sites were occupied for most daylight hours in the final month of the non‐breeding period. High levels of occupancy at the colony in the non‐breeding season have also been found for guillemots breeding in other colonies at the southern edge of their distribution in the east Pacific and Atlantic (Manuwal et al., 2001; Sinclair, 2018). Our findings demonstrate that a key motivation was the defense of higher‐quality breeding sites, which were occupied earlier, more frequently and for longer than sites of lower quality. In turn, the return date, frequency, and time spent occupying the site were highly correlated with one another. Together, these results provide strong support for the site defense hypothesis. Defense of breeding sites prior to the breeding season, and increased defense‐like behaviors linked to site quality have been well documented in avian species (white‐throated dippers *Cinclus cinclus*: Crowther et al., 2018; black kites *Milvus nigrans*: Sergio & Newton, 2003). However, few studies have demonstrated the link between site quality and time investment. One likely cause for this paucity of similar findings is the logistical challenge of collecting these data; guillemot breeding sites are easy to find, densely clustered and are readily observed without causing disturbance. Northern fulmars *Fulmarus glacialis* share these traits and, accordingly, a link between site quality and non‐breeding site occupancy has been well documented in the species (MacDonald, 1980).

Timing of return to breeding sites in the prebreeding period is an established predictor of timing of breeding (Morrison et al., 2019) and success (Aebischer et al., 1996), but what has been less clear is the role of non‐breeding season occupancy. Although the occupancy frequency was the most important factor in determining timing of breeding, we show that return date is an important indicator of future breeding success. Thus, our results extend and support a previous study in our study population which found that sites that were occupied more often in the early part of the following non‐breeding season were more successful in the subsequent breeding season (Harris & Wanless, 1989). Furthermore, while there was a relatively narrow window in which the majority of sites were first occupied, the likelihood of breeding successfully declined sharply with return rate. It may well be that although there are benefits of returning as early as possible, the individuals returning very late have markedly reduced breeding success, presumably as a result of those individuals having to occupy a site of comparatively poorer quality even if they occupy sites frequently or for long periods later in the non‐breeding season. The importance of the timing of return is further strengthened by our finding that an earlier return received equal or greater support as the indirect or direct effects of timing of breeding in predicting future breeding success. That non‐breeding occupancy of breeding sites up to 7 months prior to breeding may be as strong a predictor of breeding success as a well‐established measure such as timing of breeding, Hatchwell (1991) suggests the significance of this behavior on reproductive success.

In light of the clear associated benefits of earlier and more frequent site occupancy, the question arises of why more individuals do not undertake this behavior. Presumably, this behavior may come at some cost. Individuals that occupy breeding sites may incur increases in energetic expenditure from the need to commute between the colony and foraging and resting sites at sea. This may be particularly important in guillemots that have high flight costs (Davies & Houston, 1981). The two periods of the non‐breeding season when average level of occupancy declined supports this assertion. These decreases in occupancy overlap with periods of the year when energetic costs are expected to be high due to poor weather conditions, relatively short day lengths and, coinciding with the first decrease in occupancy in December, a partial molt of head and neck feathers that this species undertakes (Dunn et al., 2020; Harris & Wanless, 1990). Thus, individuals may be constrained from incurring the additional cost of occupying colonies at that time (Schmaljohann & Naef‐Daenzer, 2011). In the same way, individuals in poorer body condition may be constrained to limit the energy they can invest in site occupancy. Such individuals may have less capacity to manage the space and time constraints that site occupancy is likely to involve. Those individuals that occupy sites may need to obtain their daily food requirements closer to the colony, which could be suboptimal compared with areas further from the colony so providing less energy and/or nutrition, and have less time to forage because a portion of the day is spent at the colony or commuting. Consequently, individual quality may also influence site occupancy. Future research quantifying non‐breeding distribution and behavior of individuals in relation to occupancy will be required to elucidate the causes of variation in occupancy between individuals including the importance of individual quality.

Where individual constraints limit occupancy, there may be a mechanism by which this could be partially mitigated by strategies of occupation by breeding pairs, since it is likely that only one of the two is required to occupy the site at any one time to defend it. We found no independent effect of occupancy by both members of a pair on either breeding timing or breeding success. Furthermore, we found that the same effects of occupancy on breeding measures held when only one individual was present at a site, in comparison with our main analysis that did not distinguish between the presence of one or two individuals. As such, site defense would appear not to depend on the joint occupancy of partners, and this may allow one member of the pair more time for other activities such as foraging and resting, or may be experiencing a period of poor condition and is unable to occupy at a particular time. A further consideration, however, is that the time that members of a breeding pair spend together may be important for future breeding success, if pair bonding or other important social functions strengthen with time spent together. However, in contrast to other studies (Ausband, 2019; Hunter, 1999), our findings suggest this is not the case. Instead, pairs may coordinate their occupancy to maximize the time the breeding site is defended, minimize energetic costs of this behavior and spend sufficient time together to maintain the pair bond (Gwinner et al., 1994); however, individual level data will be required to confirm this.

In conclusion, we demonstrate that the opportunity to occupy a high‐quality breeding site appears to influence behavior up to 7 months prior to breeding. Those individuals that are able to defend high‐quality sites earlier and more frequently over the non‐breeding season may see associated benefits through an advanced timing of breeding and increased breeding success. Conversely individuals or pairs that are unable to occupy breeding sites may incur a decrease in key fitness measures. Further studies are required to test the generality of these findings in other populations and/or species where individuals also invest in site occupancy outside the breeding season. Non‐breeding behaviors such as non‐breeding occupancy of breeding sites thus merit greater attention and incorporation into studies exploring the drivers of demographic trends of populations.

## AUTHOR CONTRIBUTIONS

**Sophie Bennett:** Conceptualization (equal); data curation (equal); formal analysis (lead); investigation (lead); methodology (equal); project administration (equal); visualization (lead); writing – original draft (lead); writing – review and editing (equal). **Mike P. Harris:** Conceptualization (equal); data curation (equal); investigation (equal); methodology (equal); validation (equal); writing – review and editing (equal). **Sarah Wanless:** Conceptualization (equal); investigation (equal); methodology (equal); validation (equal); writing – review and editing (equal). **Jonathan A. Green:** Conceptualization (equal); investigation (equal); methodology (equal); supervision (equal); validation (equal); writing – review and editing (equal). **Kate R. Searle:** Formal analysis (supporting); investigation (equal); validation (equal); writing – review and editing (equal). **Mark A. Newell:** Data curation (equal). **Francis Daunt:** Conceptualization (equal); funding acquisition (lead); investigation (equal); methodology (equal); project administration (equal); resources (lead); supervision (lead); validation (equal); writing – review and editing (equal).

## CONFLICT OF INTEREST

The authors declare no conflict of interests.

## Figures and Tables

**FIGURE A1:**
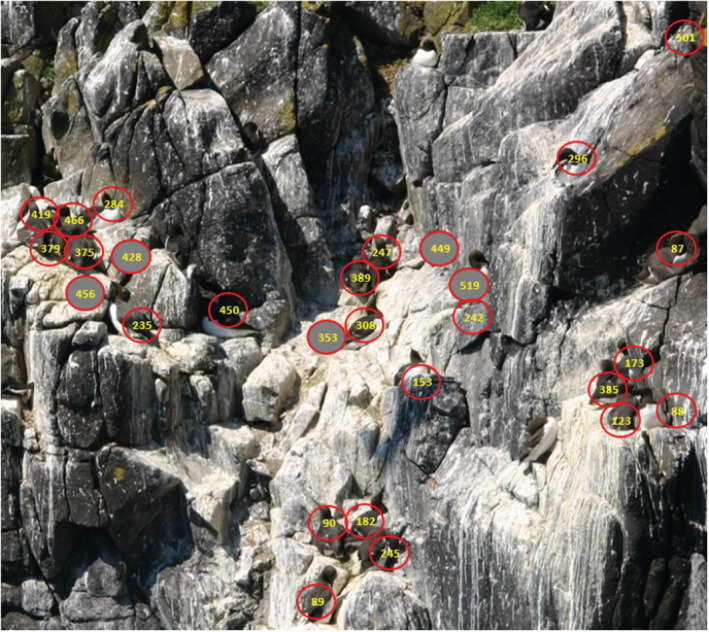
Site locations for subcolony 1

**FIGURE A2:**
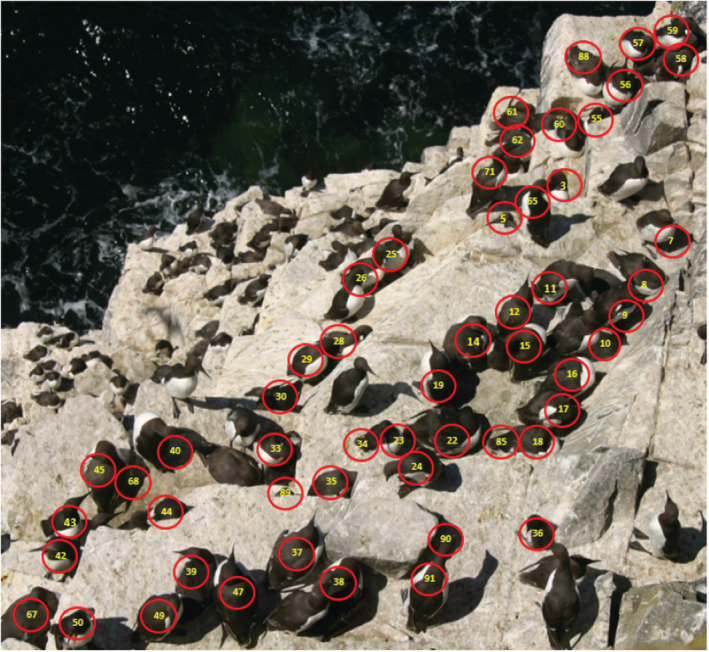
site locations for subcolony 2

**FIGURE A3:**
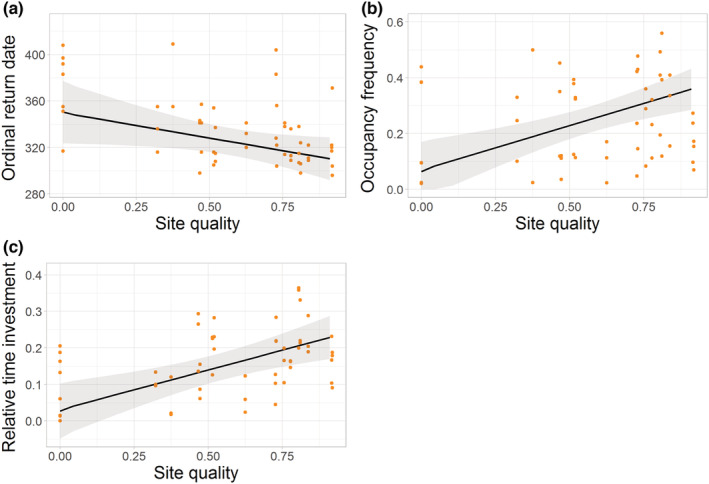
general linear model predictions, showing the relationship between a breeding site's quality and (a) return date, (b) the occupancy frequency, and c) the relative time investment at a site by a pair. Model predictions are shown as solid black lines, associated 95% confidence intervals are shown as shaded regions. Points indicate raw data.

**FIGURE A4:**
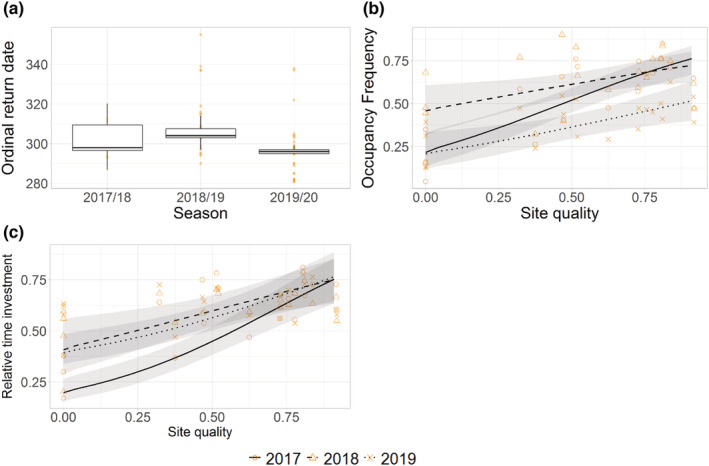
Season‐specific differences in (a) the first date a site was occupied, and relationship between site quality and (b) occupancy frequency and (c) relative time investment (length of occupancy). Raw data (points) and GLMM model predictions (fitted line ±95% CI). *N* = 59.

## Data Availability

The data used in this study are currently in the process of being deposited in the EIDC. https://doi.org/10.5285/40d28d0b‐f93d‐4c6c‐90ef‐97a26a510f81.

## APPENDIX 2

### CAMERA TECHNICAL SETUP

We used Neewer© LCD timers to trigger DSLR Canon EOS 600D cameras (Canon Inc.) to take an image every 15 or 30 min (S3) from the 1st October to the 31st March in each study year (2017/18, 2018/19, and 2019/20). To prevent water‐damage and corrosion we housed cameras and timers in waterproof PELI 1150© cases (Peli Products Limited). To accommodate the camera lens when the camera was fixed in its photo‐capture position a hole was cut in the front of the PELI© case and a section of plastic tubing with a Perspex cover was glued in place over the hole. We then housed the camera and PELI© case for subcolony 1 inside the hide used by observers when recording breeding observations for this subcolony. We secured the camera and PELI case for subcolony 2 with scaffolding adjacent to the vantage point used by the observer to make breeding observations. To include all breeding sites in frame, we adjusted the magnification and focus as required: the subcolony 1 camera was fitted with a 70–300 mm lens, the subcolony 2 camera with an 18–55 mm lens. Power to the cameras was supplied from a 12 V car battery housed externally. We exchanged camera SD cards, and checked battery levels of cameras and timers at a minimum of once a month.

## APPENDIX 3

### COMPARABILITY OF OCCUPANCY BETWEEN DIFFERENT PHOTOGRAPH FREQUENCIES

In 2017/18, we programmed the camera in subcolony 1 to take an image every 30 min, while in 2018/19 and 2019/20, we programmed the cameras in both subcolonies to take images every 15 min. Increasing the sampling frequency reduces the likelihood of missing a site being occupied. However, it could potentially make standardized comparisons of return dates, the proportion of number of days a site was occupied, and relative time investment at a site between subcolonies/non‐breeding seasons problematic if an appreciable number of site visits have durations of less than 30 min.

To investigate this possibility, we calculated the return date, the first date that a site was occupied in the non‐breeding period, the occupancy frequency of a site, the proportion of survey days that a site was occupied, throughout the non‐breeding season and the relative time investment at a site, the proportion of images on each day that a site was occupied (for those images with one or more birds present), for all data for 2018/19 and 2019/20 using the 15‐min sampling frequency, that is, all the images, and then repeating the process resampling every other image, the equivalent of sampling every 30 min. Before testing for differences in occupancy measures between the sampling frequencies, all measures were mean‐centered and scaled for each subcolony and year. Using paired t‐tests, we found no significant effect of sampling frequency on the date that a site was first occupied (*t*_df = 122_ = 0, *p* = 1) see Table A1.

We did find that sampling frequency had an effect on the occupancy frequency, and the relative time investment at a site such that the occupancy frequency higher when using a higher sampling frequency, see Table A1 (occupancy frequency: *t*_df = 103_ = 17.62, *p* < .01, relative time investment: *t*_df = 103_ = 10.58, *p* = <.01). However, the effect size of this was comparatively small (3%, and 5% respectively). Furthermore, both of these measures were very highly correlated between the two sampling frequencies (Pearson's product–moment correlation: occupancy frequency: cor = 0.99_df = 102_, *p* < .01, relative time investment: cor = 0.89_df = 102_, *p* < .01). So while there may be some minor difference in the magnitude of these values between the two sampling frequencies, we are confident that they remain comparable within a sample. As a result, we accounted for any differences in the proportion of time sites were attended and the relative time investment at sites by including a random term of “Year” in any models where both of these measures were used.

**TABLE A1:** Results from paired *t*‐tests testing the difference between three occupancy measures calculated from images taken every 15 min and every 30 min.

Occupancy measure	Mean difference	95% CI	*T*‐value	Degrees of freedom
Return date	0.0	0.00, 0.00	0	122
Occupancy frequency	**0.03**	**0.03, 0.04**	**17.62**	**103**
Relative time investment	**0.05**	**0.04, 0.07**	**10.58**	**103**

*Note*: Measures that differed significantly between image frequencies, that is, those with 95% confidence intervals not overlapping zero, are in bold.

## APPENDIX 4

### GAPS IN CAMERA DATA

**TABLE A2:** Dates of missing non‐breeding occupancy data, and the number of monitored sites affected, for study subcolonies in 2019/20.

Subcolony	Period lost	Number of days	No. sites affected
1	4th November–26th November	23	17
	18th December–17th January	31	All
	26th January–3rd February	9	All
2	12th November–17th January	66	All

As a result of the data gaps listed in Table A2, we were unable to record complete occupancy for all sites in 2019/20. We do not think that this loss of data will have had a large effect on our recording of return dates for sites or pairs as we had been able to record these metrics for the majority of sites prior to the fault: subcolony 1, first return to site = 28/29, first return of pair = 23/29, Subcolony 2, first return to site = 35/36, first return of pair = 34/36. As the other two measures of occupancy (frequency and relative time investment) were proportional we are also confident that these data gaps will not have adversely affected the data collected.

## APPENDIX 5

### 2020 BREEDING OBSERVATION METHODOLOGY

The COVID‐19 pandemic prevented normal fieldwork to record laying dates and breeding success from being conducted during the 2020 breeding season. However, we were able to take advantage of our time‐lapse photography setup, and left these cameras running at both subcolonies at a sampling frequency of 15 min beyond the 30th March when we considered the non‐breeding season to have ended. From 11th June, we were able to resume visual observations on subcolony 1 enabling breeding success to be estimated directly. We were unable to carry out in‐person observations for subcolony 2; however, no images were obtained after 23rd June due to a camera malfunction, before the breeding outcome for many of the sites was known. Hence, breeding success was only available for a subset of sites at subcolony 2 in 2020.

Using the images collected for both subcolonies, we recorded laying date as either the date when an egg was first seen at a site or if an egg was not seen but where a bird was recorded in the characteristic incubating posture in every image for 24 h (Table A3). Using this method, we may have under‐recorded events where birds lost eggs very soon after laying. However, due to the high frequency of camera images, we believe the incidence of this will have been minimal. The camera‐based assessments of laying dates were carried out by the same observers as the non‐breeding season site occupancy data, that is, MPH for subcolony 1 and SB for subcolony 2.

**TABLE A3:** Number of sites monitored directly and using time‐lapse photography to record laying and hatching dates in 2020

Subcolony	Breeding observations					
	Lay date		Hatching date		Success	
	In‐person	Camera method	In‐person	Camera method	In‐person	Camera method
1	–	29	29	–	29	–
2	–	50	–	45	–	19

We then used the hatching dates for subcolony 1 obtained by the usual visual methods, that is, the presence of shell from a hatched egg and/or the “drooped wing” posture of a brooding bird. For subcolony 2, we were unable to carry out direct observations during the chick‐rearing period, so we continued to estimate hatch dates and breeding success from time lapse images. For hatching date, we used the same criteria for in‐person observations. This assignment was then confirmed by a chick being easily visible at that site c. 3 days later. We excluded any sites from our analysis where the camera view was not sufficient to observe a change in posture or presence of egg shell, that is, the parent's body was not fully in view. As chicks grew, we were often able to see them in the images.

We also used the hatching dates obtained from subcolony 1 to verify our methodology for recording lay dates remotely. The mean incubation period in guillemots is 33.6 ± 0.05 (Harris & Wanless, 1988) days, so we subtracted 34 from each hatch date in subcolony 1 to test whether this tallied with the lay date determined from the images. If the lay date fell outside of this estimation by >2 days, we then corrected the lay date. Only two of the 29 breeding records required minor adjustment giving us confidence that the camera method for estimating laying date was robust.

In both subcolonies, we scored a site as having a successful breeding event if a chick survived to at least 15 days after hatching. Due to the camera malfunction in subcolony 2, we were unable to obtain breeding outcomes for sites that had not either failed, were still incubating an egg, had successfully fledged a chick, or had a chick present at site that was at least 15 days old by 23rd June. As a result we have a reduced sample size for breeding success for subcolony 2 in 2020 to 19/59 sites. Again, we provide the sample size of size of sites for both of these categories for each subcolony in Table A3.

## APPENDIX 6

### SITE OCCUPANCY OF PAIRS

We repeated out main analysis using equivalent pair‐level measures for all occupancy measures to determine whether any effects of site occupancy on breeding solely operated when two birds from a pair simultaneously occupied sites.

We recorded equivalent occupancy measures when sites were occupied by two birds as we did for when sites were occupied by one or two birds. These occupancy measures were as follows: The first date that a site was occupied by two birds, return date (pair), the proportion of survey days where two birds were present, occupancy frequency (pair), and the time spent occupying a site while other birds were present (the number of images on each day that a site was occupied by two birds divided by the number of images on each day where at least one bird was present in the subcolony), relative time investment (pair). As with our main analysis, we mean‐centered values within each subcolony and year prior to modeling. In all models, we included a random effect of “Subcolony Year,” except where indicated otherwise, to account for the interannual variation in extrinsic effects that may affect occupancy and breeding and inter‐subcolony variation in occupancy. We present these results in full to give a complete account of any differences from our main analysis.

80% of sites were first occupied by a pair by December 22nd (ordinal date = 357). The mean return date for a pair was November 25th ± 3 days (ordinal date = 330), and on average, sites were first occupied by a pair 29 ± 10 days after the initial visit. Sites were occupied by a pair with an average occupancy frequency of 22 ± 18% during the non‐breeding season. On average, pairs were present for 13 ± 9% of the time sites were occupied during the non‐breeding season, see Table A4.

**TABLE A4:** Average return dates, the occupancy frequency, and the relative time investment by pairs for both subcolonies across all study years

Return date			Proportion of days attended	Relative time investment
Mean	Earliest	Latest		
November 25th ± 10 (33)	October 8th (282)	March 3rd (63)	0.22 ± 0.18	0.13 ± 0.09

*Note*: Dates are calendar dates with ordinal dates in brackets.

### COMPARABILITY OF OCCUPANCY BETWEEN DIFFERENT PHOTOGRAPH FREQUENCIES

As for the data in our main analysis, we tested whether the different sampling regime in 2017 (images every 30 min, as opposed to every 15 min) affected the occupancy measures we calculated for pairs. We found no significant difference in the return date calculated using images every 15 min, compared with those using images from every 30 min; return date, *t*_df = 109_ = 0, *p* = 1, Table A5.

However, we did find the calculated occupancy frequency that a site was occupied was higher (*t*_df = 99_ = 11.66, *p* = <.01) as was the relative time investment (*t*_df = 102_ = 6.56, *p* = <0.01) at the 15 min sampling frequency. The effect size of these differences were comparatively small (3% and 2% respectively). Both of these measures were also very highly correlated between the two sampling frequencies: one or more birds (Pearson's product–moment correlation: occupancy frequency: cor = 0.98_df = 98_, *p* < .01, relative time investment: cor = 0.96_df = 102_, *p* < .01). This indicated that the relative time investments were similar. Thus, we were confident that site occupancy measures from the 15 and 30 min sampling frequencies were comparable. As a result, to include the highest resolution data we included the full dataset for pair‐level occupancy measures in the following analysis.

**TABLE A5:** Results from paired *t*‐tests testing the difference between three occupancy measures calculated from images taken every 15 min, and every 30 min.

Occupancy measure	Mean difference	CI	*T*‐value	Degrees of freedom
Return date	0	0.00, 0.00	0	109
Occupancy frequency	**0.03**	**0.03, 0.04**	**11.66**	**99**
Relative time investment	**0.02**	**0.01, 0.02**	**6.56**	**102**

*Note*: Measures that differed significantly between image frequencies, those with 95% confidence intervals not overlapping zero, are in bold.

### ASSOCIATIONS BETWEEN OCCUPANCY MEASURES

As in the main analysis, we tested the relationship between our occupancy measures to determine whether pairs that return earlier also attend more frequently and for longer. We tested these using two general linear mixed‐effects models, in the first model, we tested the effect of return date on the occupancy frequency for a site. In the second model, we tested the effect of return date and the occupancy frequency on the relative time investment at a site.

We found that, as in our main analysis, sites that were occupied earlier by pairs were occupied more frequently, estimate = −0.03, 95% CI = −0.049, −0.01, and for longer each day, estimate = −0.02, 95% CI = −0.01, −0.029; days, estimate = 0.073, 95% CI = 0.064, 0.083. The positive effect of earlier and more frequent occupancy were intensified when sites were occupied both earlier and more frequently (estimate = 0.00094, 95% CI = 0.0034, 0.015).

### OCCUPANCY AND BREEDING

We then tested whether pair occupancy measures in the non‐breeding season also affected lay date and breeding success. As with our main analysis, we predicted that higher‐quality sites would be occupied earlier and more frequently. Following from this, those sites that are occupied by a pair earlier and for longer will have an earlier lay date and higher breeding success.

In these models, we used the same error and variable structure as for the equivalent tests in the main analysis with the equivalent pair level occupancy measures.

Overall, we found no effect of any pair‐level occupancy measures on either lay date or breeding success.

### RESULTS

#### Site quality

Sites of higher quality were first occupied by pairs earlier (model 1 estimate = −13.4, 95% CI = −25.77, −1.1, Table A6, 1), for a higher occupancy frequency (model 2 estimate = 0.09, 95% CI = 0.04, 0.13, Table A6, 2), and had a higher relative time investment (model 3 estimate = 0.07, 95% CI = 0.03, 0.1, Table A6, 3) than sites of lower quality. So, we find some evidence that pairs return to the colony in the non‐breeding season to occupy and defend high‐quality breeding sites (Figure A3).

**TABLE A6:** Linear mixed‐effects model outputs assessing the effect of site quality on the date that a site was first occupied, the occupancy frequency, and the relative time investment by a pair

Model	Response variable	Fixed effects	Estimate	Standard error	Confidence interval
1	Return date (pair)	**Intercept**	**326**	**5.95**	**315.22, 337.71**
		**Site quality**	**−13.4**	**6.53**	**−25.77, −1.1**
		**Year**			
		**2018/19**	**21.4**	**6.94**	**8.26, 34.62**
		**2019/20**	**13.91**	**7.07**	**0.41, 27.28**
		Site quality*2018/19	−8.33	7.66	−22.95, 6.14
		Site quality*2019/20	−4.51	7.71	−19.16, 10.11
*Marginal R* ^*2*^ *= .36, Conditional R* ^*2*^ *= .56, n = 59*					
2	Occupancy frequency (pair)	**Intercept**	**0.21**	**0.02**	**0.17, 0.25**
		**Site quality**	**0.09**	**0.02**	**0.04, 0.13**
		**Year**			
		**2018/19**	**0.12**	**0.02**	**0.08, 0.17**
		**2019/20**	**−0.14**	**0.02**	**−0.19, −0.1**
		**Site quality*2018/19**	**−0.1**	**0.03**	**−0.15, −0.05**
		**Site quality*2019/20**	**−0.07**	**0.03**	**−0.12, −0.02**
*Marginal R* ^*2*^ *= .13, Conditional R* ^*2*^ *= .13, n = 59*					
3	Relative time investment (pair)	**Intercept**	**0.15**	**0.02**	**0.11, 0.18**
		**Site quality**	**0.07**	**0.02**	**0.03, 0.1**
		Year			
		2018/19	0.02	0.02	−0.02, 0.05
		2019/20	−0.00	0.02	−0.04, 0.03
		Site quality*2018/19	−0.02	0.02	−0.06, 0.01
		Site quality*2019/20	−0.03	0.02	−0.07, 0.01
*Marginal R* ^*2*^ *= .27, Conditional R* ^*2*^ *= .64, n = 59*					

*Note*: For “year” variables, 2017/18 was used as a reference level. The residual deviance in all models was 55, *n* years = 3. Significant terms, those with 95% confidence intervals not overlapping zero, are in bold.

#### Lay date

We found no evidence that earlier and more frequent occupancy by a pair results in an earlier lay date; no occupancy measures were retained in the best supported model (Table A7).

#### Breeding success

The simplest model with the most model support did not indicate that any pair occupancy measure had an effect on breeding success (Table A8).

From these results, we find no evidence that pairs that attend the colony earlier and spend more time at the colony together benefit from an earlier lay date and more successful breeding.

#### Model AIC tables (pairs)

**TABLE A7:** AIC table of generalized linear mixed‐effects models with different fixed effect term structures to investigate the relationship between laying date and three non‐breeding pair‐level occupancy measures.

Fixed effects structure	Number of parameters	AIC	∆AIC
**Null**	**4**	**704.07**	**0**
Relative time investment	5	705.61	1.54
Occupancy frequency	5	705.76	1.69
Return date	5	706.04	1.97
Occupancy frequency + relative time investment	6	707.32	3.25
Occupancy frequency + return date	6	707.53	3.46
Return date + relative time investment	6	707.60	3.53
Occupancy frequency*relative time investment	7	708.97	4.90
Occupancy frequency + return date + relative time investment	7	709.07	5.0
Return date*relative time investment	7	709.44	5.37
Occupancy frequency*return date	7	709.62	5.55
Return date + Occupancy frequency*relative time investment	8	710.67	6.60
Occupancy frequency + return date*relative time investment	8	710.79	6.72
Occupancy frequency*return date + relative time investment	8	711.19	7.12
Occupancy frequency*relative time investment + return date*relative time investment	9	712.27	8.20
Occupancy frequency*return date + return date*relative time investment	9	712.64	8.57
Occupancy frequency*return date + Occupancy frequency*relative time investment	9	712.87	8.80
Occupancy frequency*relative time investment + return date*relative time investment + Occupancy frequency*return date	10	714.01	9.94
Occupancy frequency*return date*relative time investment	11	716.05	11.98

*Note*: The most supported model with the simplest model structure is shown in bold.

**TABLE A8:** AIC table of generalized linear mixed‐effects models with different fixed effect term structures to investigate the relationship between breeding success and three non‐breeding pair‐level occupancy measures.

Fixed effects structure	Number of parameters	AIC	∆AIC
**Null**	**3**	**154.56**	**0**
Relative time investment	4	154.75	0.19
Return date	4	155.98	1.42
Occupancy frequency	4	156.59	2.02
Occupancy frequency*return date	6	156.63	2.06
Return date + relative time investment	5	156.64	2.08
Occupancy frequency + relative time investment	5	156.78	2.22
Occupancy frequency + return date	5	157.85	3.29
Return date*relative time investment	6	158.29	3.72
Occupancy frequency*Return date + relative time investment	7	158.61	4.04
Occupancy frequency + Return date + relative time investment	6	158.83	4.26
Occupancy frequency*relative time investment	6	158.99	4.42
Occupancy frequency + return date*relative time investment	7	160.53	5.97
Occupancy frequency*Return date + Occupancy frequency*relative time investment	8	160.84	6.27
Occupancy frequency*return date + return date*relative time investment	8	160.90	6.33
Return date + Occupancy frequency*relative time investment	7	161.07	6.50
Occupancy frequency*relative time investment + return date*relative time investment	8	162.76	8.19
Occupancy frequency*relative time investment + return date*relative time investment + Occupancy frequency*return date	9	163.16	8.59
Occupancy frequency*return date*relative time investment	10	165.33	10.76

*Note*: The most supported model with the simplest model structure is shown in bold.

## APPENDIX 7

### SITE OCCUPANCY BY ONE BIRD

We also tested whether any relationships between occupancy and site quality and breeding timing and success were different when using equivalent occupancy measures when just one bird was present. In these tests, we found no significant differences from our main analysis. We briefly summarize these results below.

Summary occupancy measures for one bird shared the same mean date of first occupancy as the analysis using one or more bird occupancy measures. However, the frequency and length of attendance was shorter. The mean date that a site was first occupied by one bird was October 27th ± 11.7 days (OD = 297). Sites were occupied for an average of 54 ± 19% of days, and for 42 ± 14% of the time that a subcolony was occupied. The equivalent measures for one or more bird occupancy measures were as follow: The mean date that a site was first occupied was October 27th ± 11.7 days (OD = 297). Sites were occupied for an average of 46 ± 18% of days, and for 55 ± 15% of the time that a subcolony was occupied.

As with the main analysis, sites occupied earlier were also occupied more frequently (estimate = −0.02, 95% CI = −0.04, −0.01). Those sites that were occupied earlier, or more frequently were also occupied for longer (return date, estimate = −0.20, 95% CI = −0.24, −0.16, days, frequency estimate = 0.45, 95% CI = 0.41, 0.50). There was no clear difference in this comparison between occupancy measures from those we undertook in the main analysis.

Supporting our first hypothesis, sites of higher quality were occupied earlier (estimate = −4.67, 95% CI = −9.72, −0.43) more frequently (estimate = 0.79, 95% CI = 0.51, 1.08), and for longer (estimate = 0.79, 95% CI = 0.52, 1.06). These results again show no clear difference from our main analysis, although the relationship between site quality and how long sites were occupied was somewhat weaker.

Mirroring our main analysis, there was weak support for hypothesis 2a that sites occupied more frequently had an earlier lay date (estimate = −0.88, 95% CI = −1.85, −0.09). Furthermore, in support of hypothesis 2b, sites were more likely to be successful when they were occupied earlier (estimate = −0.5, 95% CI = −1.06, −0.12).

### MODEL AIC TABLES (ONE BIRD PRESENT) (TABLES A9 AND A10)

**TABLE A9:** AIC table of generalized linear mixed‐effects models with different fixed effect term structures to investigate the relationship between laying date and three non‐breeding occupancy measures where one bird was present at a site.

Fixed effects structure	Number of parameters	AIC	∆AIC
**Occupancy frequency**	**5**	**709.88**	**0**
Occupancy frequency + return date	6	710.42	0.54
Occupancy frequency + return date + relative time investment	7	710.71	0.83
Occupancy frequency + relative time investment	6	710.92	1.04
Null	4	711.27	1.39
Return date	5	711.32	1.44
Occupancy frequency*return date	7	711.33	1.44
Occupancy frequency*return date + relative time investment	8	711.94	2.06
Return date + Occupancy frequency*relative time investment	8	712.07	2.19
Occupancy frequency*relative time investment	7	712.26	2.38
Occupancy frequency + return date*relative time investment	8	712.41	2.52
Relative time investment	5	712.97	3.09
Occupancy frequency*return date + Occupancy frequency*relative time investment	9	713.06	3.18
Return date + relative time investment	6	713.16	3.28
Occupancy frequency*return date + return date*relative time investment	9	713.54	3.66
Occupancy frequency*relative time investment + return date*relative time investment	9	713.71	3.83
Return date*relative time investment	7	714.46	4.58
Occupancy frequency*relative time investment + return date*relative time investment + Occupancy frequency*return date	10	714.70	4.82
Occupancy frequency*return date*relative time investment	11	716.0	6.11

*Note*: The most supported model with the simplest model structure is shown in bold.

**TABLE A10:** AIC table of generalized linear mixed‐effects models with different fixed effect term structures to investigate the relationship between breeding success and three non‐breeding occupancy measures where one bird was present at a site.

Fixed effects structure	Number of parameters	AIC	∆AIC
**Return date**	**4**	**152.17**	**0**
Occupancy frequency + return date	5	152.80	0.62
Return date + relative time investment	5	154.30	2.13
Occupancy frequency + return date + relative time investment	6	154.63	2.46
Occupancy frequency	4	154.77	2.59
Occupancy frequency*return date	6	154.90	2.72
Return date*relative time investment	6	155.26	3.09
Null	3	155.65	3.47
Occupancy frequency + return date*relative time investment	7	156.01	3.84
Return date + Occupancy frequency*relative time investment	7	156.62	4.45
Relative time investment	4	156.78	4.60
Occupancy frequency*return date + relative time investment	7	156.82	4.64
Occupancy frequency + relative time investment	5	156.94	4.76
Occupancy frequency*relative time investment + return date*relative time investment	8	157.88	5.70
Occupancy frequency*return date + return date*relative time investment	8	158.19	6.02
Occupancy frequency*Return date + Occupancy frequency*relative time investment	8	158.77	6.59
Occupancy frequency*relative time investment	6	158.88	6.71
Occupancy frequency*relative time investment + return date*relative time investment + Occupancy frequency*return date	9	160.15	7.98
Occupancy frequency*return date*relative time investment	10	161.29	9.12

*Note*: The most supported model with the simplest model structure is shown in bold.

## APPENDIX 8

### MODEL AIC TABLES 1+

**TABLE A11:** AIC table of generalized linear mixed‐effects models with different fixed effect term structures to investigate the relationship between the return date and occupancy frequency and relative time investment at a site

Fixed effects structure	Number of parameters	AIC	∆AIC
**Return date*Occupancy frequency**	**11**	**2099.71**	**0**
Return date + Occupancy frequency	10	2118.84	19.13
Occupancy frequency	9	2123.39	23.69
Return date	8	2125.78	26.07
Null model	9	2127.09	27.39

*Note*: The most supported model with the simplest model structure is shown in bold.

**TABLE A12:** AIC table of generalized linear mixed‐effects models with different fixed effect term structures to investigate the relationship between laying date and three non‐breeding occupancy measures.

Fixed effects structure	Number of parameters	AIC	∆AIC
**Occupancy frequency**	**5**	**710.49**	**0**
Return date + occupancy frequency	6	710.89	0.41
Null	4	711.27	0.78
Return date	5	711.32	0.83
Return date + relative time investment	6	711.59	1.10
Return date + occupancy frequency + relative time investment	7	711.76	1.28
Relative time investment	5	711.77	1.29
Return date*occupancy frequency	7	712.08	1.60
Return date + relative time investment	6	712.44	1.95
Return date*occupancy frequency + relative time investment	8	713.04	2.55
Return date*relative time investment + relative time investment	8	713.16	2.70
Occupancy frequency*relative time investment	7	713.31	2.82
Return date + occupancy frequency*relative time investment	8	713.42	2.94
Return date*relative time investment	7	713.77	3.28
Return date*occupancy frequency + return date*relative time investment	9	713.85	3.36
Return date*occupancy frequency + occupancy frequency*relative time investment	9	714.74	4.25
Return date*relative time investment + occupancy frequency*relative time investment	9	714.80	4.32
Return date*occupancy frequency + return date*relative time investment + occupancy frequency*relative time investment	10	715.55	5.07
Return date*occupancy frequency*relative time investment	11	716.13	5.65

*Note*: The most supported model with the simplest model structure is shown in bold.

**TABLE A13:** AIC table of generalized linear mixed‐effects models with different fixed effect term structures to investigate the relationship between breeding success and three non‐breeding occupancy measures

Fixed effects structure	Number of parameters	AIC	∆AIC
**Return date**	**4**	**152.18**	**0**
Return date + occupancy frequency	5	153.32	1.15
Return date + relative time investment	5	153.55	1.37
Relative time investment	4	155.12	2.95
Return date*relative time investment	6	155.22	3.04
Return date + occupancy frequency + relative time investment	6	155.50	3.33
Return date*occupancy frequency	6	155.53	3.36
Null	3	155.65	3.47
Occupancy frequency	4	155.78	3.60
Return date + occupancy frequency*relative time investment	7	157.05	4.87
Occupancy frequency + relative time investment	5	157.23	5.06
Return date*relative time investment + occupancy frequency	7	157.29	5.12
Return date*occupancy frequency + relative time investment	7	157.75	5.58
Return date*occupancy frequency + return date*relative time investment	8	159.04	6.87
Return date*relative time investment + occupancy frequency*relative time investment	8	159.08	6.91
Occupancy frequency*relative time investment	6	159.19	7.02
Return date*occupancy frequency + occupancy frequency*relative time investment	8	159.30	7.12
Return date*occupancy frequency + return date*relative time investment + occupancy frequency*relative time investment	9	160.77	8.59
Return date*occupancy frequency*relative time investment	10	161.93	9.75

*Note*: The most supported model with the simplest model structure is shown in bold.

## APPENDIX 9

### RANDOM EFFECTS STRUCTURES 1+ (TABLES A13 AND A14)

**TABLE A14:** AIC table of generalized linear mixed‐effects models with different random effect term structures

Fixed effect model structure	Random effect structure			AIC	ΔAIC
	Intercept	Slope	Combined intercept + slope		
Occupancy frequency ~ Return date	X	X		1309.01^a^	n/a
			X	1310.93^a^	n/a
	**X**			**1335.21**	0
		X		1706.64	371.43
Relative time investment ~ Occupancy frequency*Return date	**X**	**X**		**2217.91**	0
			X	2219.38	1.47
	X			2505.88	287.97
		X		3372.55	1154.64
Lay date ~ Occupancy frequency	**X**			**709.96**	0
		X		711.96^a^	n/a
			X	712.84^a^	n/a
	X	X		720.08^a^	n/a
Breeding success ~ Return date			X	151.37^a^	n/a
	**X**			**153.02**	0
	X	X		154.41	1.39
		X		172.58	19.56

*Note*: The most supported model with the simplest model structure is shown in bold.

^a^ Signifies that the model could not converge, or the fit was singular indicating that the random effect structure was too complex to be supported by the data.
